# CULTIVATION OF PROGNOSTIC AWARENESS IS A TEAM TASK: PALLIATIVE PHYSICIAN AND SOCIAL WORKER INSIGHTS

**DOI:** 10.1093/geroni/igad104.2801

**Published:** 2023-12-21

**Authors:** Arden ODonnell, Judith Gonyea

**Affiliations:** Boston University, Boston, Massachusetts, United States; Boston University, Boston, Massachusetts, United States

## Abstract

For patients experiencing serious or life-limiting illnesses, informed decision-making depends on understanding prognosis and illness trajectory. Although physicians are viewed as the most appropriate group to share medical information around prognosis, there is growing recognition that other clinicians can contribute to these discussions. Indeed, cultivation of prognostic awareness (CPA) does not happen in a single conversation; it is a series of conversations in which patients continue to redefine medical treatments and broader life goals. The reframing of CPA as an iterative process, coupled with growing palliative care demands, has led to social workers’ (SWs) and nurses’ engagement, and even leadership, in goals of care (GOC) conversations. Using role theory, this qualitative study therefore explored aligned and/or disparate views on professional role boundaries in CPA through interviews with 17 SWs and 14 physicians. Our data analysis was inductive and iterative with the culminating phases being theme generation and interpretation. Key findings revealed a shared perspective by SWs and physicians that CPA is a “team task” and that SWs have essential roles as experts in assessing and supporting patients’ emotional and psychological capacity for processing prognostic information, and as boundary spanners to patients/families and physicians (i.e., translating medical information, increasing effective communication, and reinforcing a holistic perspective). Both professions identified managing patients’/families’ expectations as a shared role. SWs experienced role conflict, however, in their advocacy for patient autonomy. Factors enhancing role consensus were communication, collaboration, professional respect, experience, and trust. Findings support the trend of expanding SWs role in serious illness conversations.

